# Supplementary material for: SiGe quantum wells with oscillating Ge concentrations for quantum dot qubits
Source: Nat Commun. 2022 Dec 15;13:7777. doi: 10.1038/s41467-022-35510-z (PMC9755230; doi:10.1038/s41467-022-35510-z)
Supplement: Supplementary file 1 — Supplementary Information [file 41467_2022_35510_MOESM1_ESM.pdf]

Supplementary Information: SiGe quantum wells with oscillating Ge concentrations for quantum dot qubits

SUPPLEMENTARY NOTE 1. DETAILS OF THE EFFECTIVE MASS VIRTUAL CRYSTAL CALCULATION OF THE WIGGLE WELL VALLEY SPLITTING

The effective mass virtual crystal (EMVC) approximation calculations of the valley splitting E_v shown in Fig. 1(c) of the main text and Supplementary Fig. 1 were performed as follows. The electron is confined by a barrier and an applied electric field in the z direction. Averaging over the lateral directions gives a one-dimensional, two-component Schrödinger equation for the envelope functions $\phi_\pm(z)$ that appear in Eq. (2) of the Methods section of the main text. The equation uses the longitudinal effective mass, $m_l = 0.92m_0$, for the kinetic energy term. The diagonal intravalley potential for the model is $V(z) = V_F(z) + V_B(z) + V_{\rm osc}(z)$. The external electrostatic potential energy is given by $V_F(z) = -eFz$, where $F = 8.5$ MV/m. The barrier potential is $V_B(z) = \frac{B}{2}[1 + \tanh(z/w)]$ with the barrier height $B = 0.15$ eV and barrier width $w = 1$ nm. The Wiggle Well potential is $V_{\rm osc}(z) = n_{\rm Ge}V_0[1 - \cos(qz)]/2$, where V_0 is the difference in site energies between the s-like conduction-band levels of Si and Ge. We take this as $V_0 = -1.53$ eV from Table I of Ref. [1]. The off-diagonal intervalley potential that connects $\phi_+(z)$ to $\phi_-(z)$ has the additional factor $\exp[\pm i(K_z - K_z' - 2k_0)z]$ with the contributions of the reciprocal lattice vectors

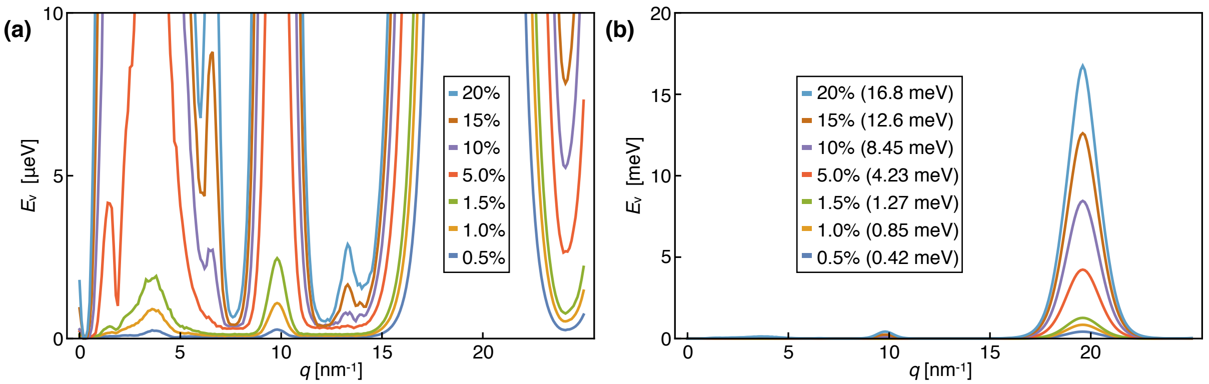

Supplementary Figure 1. The contribution to the valley splitting E_v due to a sinusoidal Ge concentration in the quantum well as a function of the wavevector q, similar to Fig. 1(c) in the main text, with a smaller scale (a) to show the low-concentration peaks at low q and a larger scale (b) to show the peaks at $q \approx 20$ nm^{-1}. The average concentration $n_{\rm Ge}$ of Ge in the quantum well of each curve is shown in the inset legend. The energy splittings listed in the inset are the maximum E_v calculated for each concentration. Source data are provided as a Source Data file.

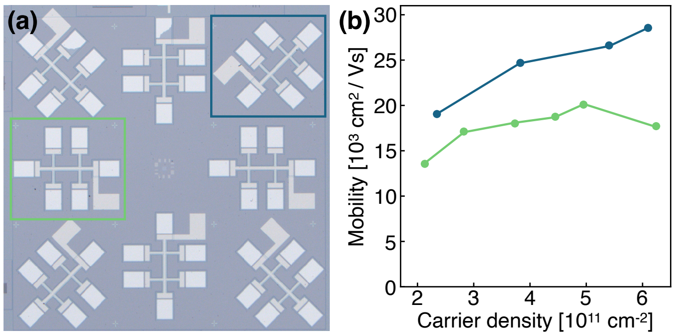

Supplementary Figure 2. Wiggle Well Hall Bars. (a) Optical image of Hall bar devices measured. The length of the Hall bar between the central ohmics is 200 µm. The Hall bar top gate is isolated from the heterostructure by 20 nm of ALD-grown aluminum oxide. All deposited metal is a Ti/Pd stack. (b) Transport mobility results of two Hall bar devices highlighted in (a), performed at ∼2 K. Source data are provided as a Source Data file.

weighted by the appropriate combinations of $c_\pm(\mathbf{K})$, the coefficients of the cell-periodic parts of the Bloch functions. These coefficients are given in Table I of Ref. [2] for bulk Si. Extinction effects in the Si lattice turn out to be extremely important for the calculation of E_v for the long-period Wiggle Well, with E_v actually vanishing at the oscillation period $\lambda_{\rm long}$ in the absence of disorder. Even when disorder is present, E_v at $\lambda_{\rm long}$ is much less than E_v at $\lambda_{\rm short}$, as seen in Fig. 1(c) of the main text and in Supplementary Fig. 1. This means that $c_+(\mathbf{K})$ must be recalculated when Ge is present. This is also done using a virtual crystal approximation in which 59 $c_\pm(\mathbf{K})$ coefficients are used [3]. The calculation requires disorder averaging, which leads to a certain amount of noise in the calculated $E_v(q)$ plots in Supplementary Fig. 1.

SUPPLEMENTARY NOTE 2. FABRICATION DETAILS AND HALL MEASUREMENT

Hall bars and quantum dot devices were fabricated simultaneously on the same ∼10 mm chip. A 15 nm layer of aluminum oxide gate dielectric is grown by atomic layer deposition (ALD) at 200 °C. This oxide is etched by dilute HF in a 30 µm square region centered around the dot region. Another 5 nm of aluminum oxide is then deposited. This results in 5 nm of deposited oxide over the dot region and 20 nm over the Hall bars. The chip then undergoes a 15 min, 450 °C forming gas anneal. The Hall bar gate metal is a bi-layer of titanium and palladium, patterned by photo-lithography. The quantum dot gate design has three layers of aluminum patterned by electron-beam-lithography. Each gate layer is isolated by the self oxidation of the aluminum, enhanced by a 15 min downstream oxygen plasma ash. Supplementary Fig. 2 shows an optical image of the Hall bars measured and

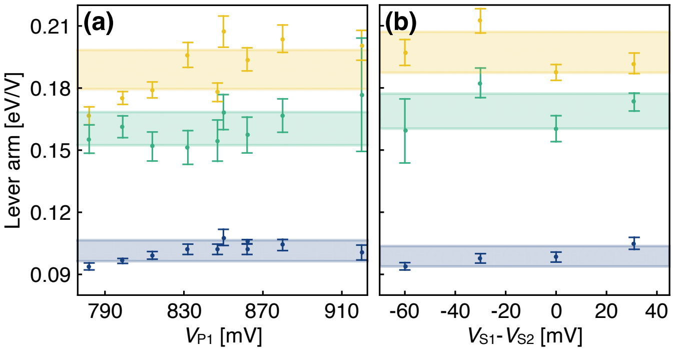

Supplementary Figure 3. Lever arms for voltage tunings. (a) Lever arms for gates S1 (yellow), S2 (green), and P1 (blue), as a function of the corresponding voltages, for the 'symmetric' voltage tuning method described in the main text. (b) Lever arms for S1 (yellow), S2 (green), and P1 (blue) of the 'asymmetric' voltage tuning method described in the main text. In both plots, the shaded regions are ±5% around the average. Error bars correspond to the standard error in the fit to Supplemental Eq. (2). Source data are provided as a Source Data file.

the transport mobility results of the measurements as a function of carrier density, measured at ∼2 K. The peak mobility reported here is 5-10 times lower than other recently reported values for pure silicon quantum wells [4–6]. However, the estimated electronic mean-free path in this device is ∼ 1 μm, so we do not expect this mobility to be a limiting factor for qubit formation or performance.

SUPPLEMENTARY NOTE 3. GATE LEVER ARMS FOR DOT TUNING

The lever arm α of the plunger gate P1 to the dot used for pulsed-gate spectroscopy is measured by thermally broadening the charge-sensed electron charging transition. The gate voltage is swept over the transition as the mixing chamber temperature is increased, and the current through the charge sensor is fit to [7]

$$I_{\mathrm{CS}}(V) = A \tanh\left[\frac{\alpha(V - V_0)}{2k_B T_e}\right] + bV + I_0, \tag{1}$$

in order to extract $\tau = T_e/\alpha$ as a function of the mixing chamber temperature T_{MC}, where k_B is Boltzmann's constant, T_e is the electron temperature, and A, b, V_0 and I_0 are additional fitting parameters. The lever arm α, as well as the base electron temperature T_{e_0}, are determined by fitting τ as a function of T_{MC} to the phenomenological expression

$$\tau = \frac{1}{\alpha}\sqrt{T_{\mathrm{MC}}^2 + T_{e_0}^2}. \tag{2}$$

For the ‘symmetric’ tuning method where both screening gates S1 and S2 are changed in the same voltage direction, the lever arm is measured at every other voltage tuning. For voltage tunings where the P1 lever arm is not explicitly measured, the average of the two nearest tunings is used. For the ‘asymmetric’ tuning method where S1 and S2 are changed in opposite directions, the lever arm is measured at every tuning. Relative lever arms between a screening gate and P1 are determined by measuring the slope of a transition line as both gate voltages are changed. Using the absolute lever arm of P1 and the relative lever arms for the screening gates, their absolute lever arms to the dot are calculated.

Supplementary Fig. 3 shows these lever arms for both the ‘symmetric’ and ‘asymmetric’ tuning methods. As shown, the lever arms for all three gates stays within 5% of the average value for most tunings. There is no noticeable difference in the lever arms between the tuning methods, despite the significant difference in valley splitting tuning. This may indicate that this method of tracking the lever arms is not a sensitive enough technique to measure the lateral movement expected in the ‘asymmetric’ tuning scheme. Our assumption that the dot remains approximately stationary for the ‘symmetric’ tuning scheme is based on a previous study of valley splitting in a device

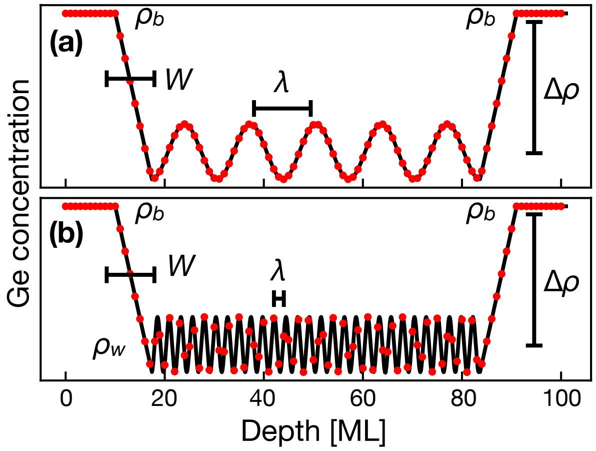

Supplementary Figure 4. Illustration of (a) the long-period Wiggle Well, and (b) the short-period Wiggle Well, simulated using NEMO-3D. Black lines represent the ideal concentration profile, and red points represent the concentrations sampled at each layer. Both wells have a linearly graded interface concentration with width $W = 1$ nm. The difference in Ge concentration between the bulk (ρ_b) and the well (ρ_w) is $\Delta\rho$, which is always fixed at 0.25 to prevent the wavefunction from spilling out of the quantum well. The concentration oscillation periods are $\lambda = 1.8$ nm in (a) and $\lambda = 0.32$ nm in (b). The amplitude of the concentration oscillation was adjusted, such that $\rho_w = A$, where A is the oscillation amplitude. Depth is reported in units of monolayers (ML). Source data are provided as a Source Data file.

with a gate structure nearly identical to the one used here [8]. In that study, the tuning scheme is identical to the 'symmetric' tuning scheme here and the dot location is determined through COMSOL simulations over the experimental tuning range. These simulations showed the center of mass of the dot remained stationary, to within 1 nm.

SUPPLEMENTARY NOTE 4. ADDITIONAL DETAILS OF NEMO SIMULATIONS

Supplementary Fig. 4 shows schematic illustrations of the Ge concentration profiles used to generate the lattice simulated in NEMO-3D. At a given layer, each atom in the lattice is assigned to be either Si or Ge, where the probability of choosing Ge is given by the average concentration in a given layer.

SUPPLEMENTARY REFERENCES

[1] Y. M. Niquet, D. Rideau, C. Tavernier, H. Jaouen, and X. Blase, Onsite matrix elements of the tight-binding Hamiltonian of a strained crystal: Application to silicon, germanium, and their alloys, Phys. Rev. B **79**, 245201 (2009).

[2] A. L. Saraiva, M. J. Calderón, R. B. Capaz, X. Hu, S. Das Sarma, and B. Koiller, Intervalley coupling for interface-bound electrons in silicon: An effective mass study, Phys. Rev. B **84**, 155320 (2011).

[3] Y. Feng and R. Joynt, Enhanced valley splitting in si layers with oscillatory ge concentration, Phys. Rev. B **106**, 085304 (2022).

[4] F. Schaffler, D. Tobben, H. J. Herzog, G. Abstreiter, and B. Hollander, High-electron-mobility si/sige heterostructures: influence of the relaxed sige buffer layer, Semicond Sci Tech **7**, 260 (1992).

[5] B. Paquelet Wuetz, P. L. Bavdaz, L. A. Yeoh, R. Schouten, H. van der Does, M. Tiggelman, D. Sabbagh, A. Sammak, C. G. Almudever, F. Sebastiano, J. S. Clarke, M. Veldhorst, and G. Scappucci, Multiplexed quantum transport using commercial off-the-shelf cmos at sub-kelvin temperatures, npj Quantum Information **6**, 43 (2020).

[6] S. F. Neyens, R. H. Foote, B. Thorgrimsson, T. J. Knapp, T. McJunkin, L. M. K. Vandersypen, P. Amin, N. K. Thomas, J. S. Clarke, D. E. Savage, M. G. Lagally, M. Friesen, S. N. Coppersmith, and M. A. Eriksson, The critical role of substrate disorder in valley splitting in Si quantum wells, Appl. Phys. Lett. **112**, 243107 (2018).

[7] L. P. Kouwenhoven, C. M. Marcus, P. L. McEuen, S. Tarucha, R. M. Westervelt, and N. S. Wingreen, Mesoscopic electron transport (Kluwer, 1997) p. 105.

[8] T. McJunkin, E. R. MacQuarrie, L. Tom, S. F. Neyens, J. P. Dodson, B. Thorgrimsson, J. Corrigan, H. E. Ercan, D. E. Savage, M. G. Lagally, R. Joynt, S. N. Coppersmith, M. Friesen, and M. A. Eriksson, Valley splittings in Si/SiGe quantum dots with a germanium spike in the silicon well, Phys. Rev. B **104**, 085406 (2021).
